# Exploration of nucleosome positioning patterns in transcription factor function

**DOI:** 10.1038/srep19620

**Published:** 2016-01-21

**Authors:** Kazumitsu Maehara, Yasuyuki Ohkawa

**Affiliations:** 1 Department of Advanced Medical Initiatives, JST-CREST, Faculty of Medical Sciences, Kyushu University, 3-1-1 Maidashi, Higashi-ku, Fukuoka, Fukuoka 812-8582, Japan

## Abstract

The binding of transcription factors (TFs) triggers activation of specific chromatin regions through the recruitment and activation of RNA polymerase. Unique nucleosome positioning (NP) occurs during gene expression and has been suggested to be involved in various other chromatin functions. However, the diversity of NP that can occur for each function has not been clarified. Here we used MNase-Seq data to evaluate NP around 258 *cis-*regulatory elements in the mouse genome. Principal component analysis of the 258 elements revealed that NP consisted of five major patterns. Furthermore, the five NP patterns had predictive power for the level of gene expression. We also demonstrated that selective NP patterns appeared around TF binding sites. These results suggest that the NP patterns are correlated to specific functions on chromatin.

The nucleosome is the basic unit of chromatin that is essential to genomic architecture in eukaryotes. The positions of the nucleosomes (nucleosome positioning; NP) are dynamically determined by transcription factors (TFs) that bind to specific DNA sequences. TF binding leads to NP alterations by chromatin remodelling, which are essential for transcriptional activation. Accordingly, NP has been shown to be correlated with transcriptional state^1^,^2^.

The digestion of chromatin with micrococcal nuclease (MNase) has been widely used to map individual nucleosomes on a locus in the genome^3^. Recent developments in deep sequencing technology in combination with MNase (MNase-Seq) have enabled high-resolution NP analysis^4^,^5^,^6^, especially in studies of budding yeast. These methods have revealed that NP follows specific patterns and have shown the existence of nucleosome depleted regions (NDR), which are thought to facilitate RNA Polymerase II (RNAP2) recruitment^7^,^8^,^9^. Albert *et al.* revealed that the TATA box and transcription start sites (TSS) reside at both ends of linker DNA in yeast^4^, suggesting NP determines the start point of transcription. Another characteristic of NP revealed by MNase-Seq is that the nucleosomes are aligned at strict and regular intervals, as shown in the binding of CTCF, an insulator binding protein that forms boundaries in the genome^10^,^11^. Recently, Ranjan *et al.* showed that yeast SWR1, a chromatin remodelling enzyme, preferentially recognizes long nucleosome-free DNA^12^; therefore, we hypothesized that there may be another structural property of chromatin that is recognized by such factors.

NP has also been suggested to be critical for transcription regulation in mammalian genomes because of the absence of core promoter sequences, which are comprehensive markers of promoter regions in yeast^13^,^14^. Determining high-resolution NP in mammalian genomes is more difficult than that in the yeast genome because mammalian genomes are much larger; therefore, MNase signal averaging^4^,^5^,^15^,^16^ has been used to overcome this difficulty. Teif *et al.* used a signal averaging method, average profiling, to demonstrate that nucleosome occupancies could change around lineage-specific TF binding sites detected by ChIP-seq (TFBSs) during the differentiation of mouse embryonic stem cells^17^. Kundaje *et al.* profiled several NP patterns at TFBSs and found that asymmetric NP is the major feature In TSSs and also in TFBSs and that the asymmetric pattern was true for histone marks but not for CTCF and DNase-I hypersensitive sites^18^. These data suggest that the diversity of NP patterns could depend on biological functions of TFs.

To explore various types of NP pattern caused by TF binding, we first collected comprehensive profiles of the average nucleosome densities (PANDs) in 258 *cis-*regulatory elements (computationally predicted potential TF binding sites) using fixed MNase-Seq data from mouse C2C12 myoblast cells. These produced wave shapes representing nucleosome detection frequency. The PANDs were summarized by principal component (PC) analysis and we identified five NP patterns that represented five major types of nucleosome distribution. These five NP patterns were correlated to gene expression and, therefore, the five NP patterns could predict expression of neighbouring genes. We also found that TF function could be predicted by the PANDs assigned to the five NP patterns.

## Results

### Fixed MNase-Seq refines NP extraction

To identify the NP patterns related to transcription activation or suppression on the whole genome, we attempted to extract NP by MNase analysis on a deep sequencing platform (Supplementary Fig. S1a) using ~10^6^ C2C12 cells undergoing skeletal muscle differentiation despite MNase-Seq normally requiring ~10^8^ cells to obtain significant NP data. We prepared cross-linked chromatin fractions before MNase digestion to obtain precise NP information. DNA was completely digested into mono-nucleosome size fragments (Supplementary Fig. S1b, mean ± s.d.: 164 ± 12 bp), because NP data are sensitive to fragment size^19^. The number of mapped reads we acquired was approximately 20 million (myoblast, 21,729,582; myotube, 19,877,072). As expected, the signal was dispersed (Supplementary Fig. S1c); for example, assuming 20 million reads, the probability that at least one read was detected within a 200 bp interval is approximately 77%, assuming a binomial distribution, a mouse genome size of 2.7 Gbp and a nucleosome repeat length of 200 bp.

**Figure 1:**
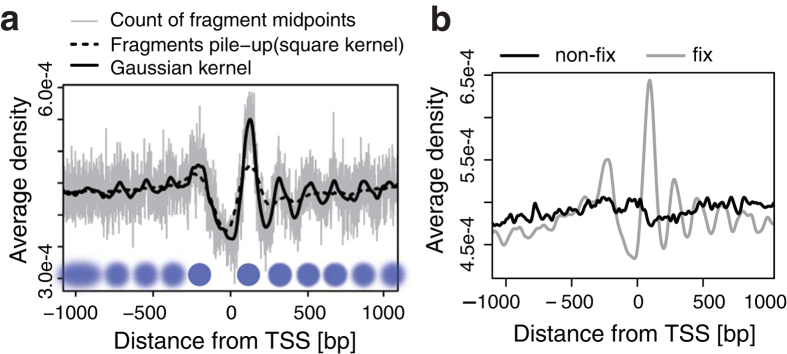
Density estimation of nucleosomes from fixed MNase-Seq. (**a**) Comparison of signal processing methods for single PAND analysis. The X-axis shows the distance (in bp) from the TSSs, and the Y-axis shows nucleosome density averaged over all genes. The grey line represents counts of fragment mid-points with 1 bp resolution. The black solid line shows these counts smoothed by a Gaussian kernel, and the dotted line represents the result of fragment pile-up in which mapped reads are extended to the fragment length and accumulated. The blue circles represent inferred positions of the nucleosome (blur: fuzzy, solid: well-positioned). (**b**) A PAND within 1 kbp of a TSS in fixed (grey) and non-fixed (black) samples. The effect of fixation was evaluated for mono-nucleosome sized samples. See Supplementary Figure S1d for fragment size. Fixed samples show clearer positioning around TSSs.

To overcome the signal dispersion for detecting NP, we applied the average profiling method, profile of the average nucleosome density or PAND, which is commonly used in ChIP-Seq and MNase-Seq studies^17^,^20^,^21^ to extract distributions of TFs or nucleosomes around anchor points of interest, e.g. gene promoters or TFBSs. We created PANDs from our MNase-Seq data for all mouse gene transcription start sites (TSSs) (Fig. 1a). The PAND wave shape indicates the average frequency (y-axis) of nucleosome detection at each position (x-axis). Well-positioned ±1 nucleosomes around TSSs were apparent in smoothed PAND data. This result is consistent with the PANDs obtained from the ChIP-Seq input data, also using C2C12 cells, generated by Asp *et al.*^22^ (Supplementary Fig. S1d). We further confirmed that clear sinusoidal PAND wave shapes around CTCF binding sites were obtained as previously reported (Supplementary Fig. S1e)^11^. These data suggest that the PANDs could visualize NP from its wave shape. To evaluate if fixation affected the efficiency of NP detection, we compared PANDs for all gene TSSs using fixed (cross-linked) and non-fixed (without cross-linking) MNase-Seq (Fig. 1b and Supplementary Fig. S1f). Although the PANDs without fixation lost clear peaks, the PANDs with fixation showed clear peaks especially for TSSs at ±1 nucleosome, even with 20 million reads (~0.006 reads/bp), which is 1/10th of the depth usually generated for mammalian MNase-Seq datasets^23^. We therefore concluded that the combination of fixation and PAND enabled the detection of NP with the limited reads number generally generated in ChIP-Seq.

### Five common NP patterns in C2C12 cells

**Figure 2:**
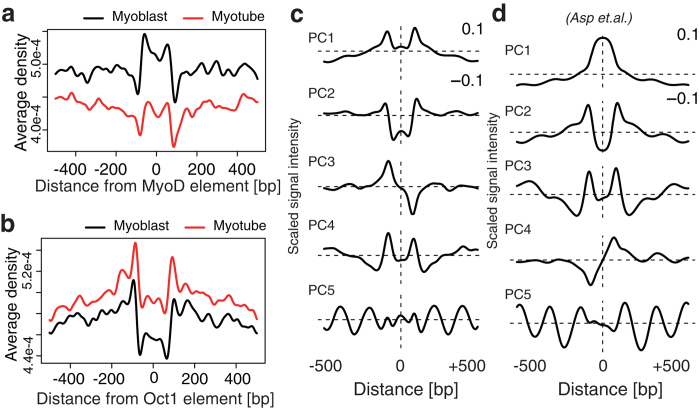
Five components extracted from PAND shapes around *cis-*regulatory elements. (**a**,**b**) PANDs of MyoD (**a**) and Oct (**b**) within 500 bp of their *cis-*regulatory elements. The X-axis denotes the distance (in bp) from the origin of the centre of a *cis*-regulatory element, and the Y-axis denotes the nucleosome density averaged over all *cis-*regulatory elements. (**c**,**d**) Principal components of the 258 PANDs. (**c**) The 1st to 5th principal components (top to bottom) were ordered by the contribution ratio and contributed approximately 80% of the cumulative contribution ratio. (**d**) The principal components of the ChIP-Seq input data from Asp *et al.*^22^.

PANDs of CTCF binding sites and TSSs produced clear and similar wave shapes from different data sets so we hypothesized that each TF could also have a unique NP. Therefore, we assessed PANDs around known TF binding sites using a *cis*-regulatory element database from TRANSFAC^24^. The PANDs around 258 *cis-*regulatory elements were obtained from the fixed MNase-Seq data (Supplementary Fig. S2). Representative data of the MyoD *cis*-regulatory element (MYOD_Q6) showed increased nucleosome occupancy around its binding sequence (Fig. 2a). In contrast, the Oct1 *cis*-regulatory element (OCT1_07) had decreased nucleosome occupancy near its binding sequence (Fig. 2b). However, we could not categorize these PANDs from their shape. Therefore we further attempted to extract common shapes from the PANDs.

To extract common wave shapes across the PANDs shown in Supplementary Figure S2, we employed principal component (PC) analysis. The each column of matrix consisted of a PAND that expresses one base resolution of the signal intensity around a specific *cis*-regulatory element. Therefore, each PAND shape could be treated as the weighted sum of PCs that is common to the wave shape in PANDs. The result showed that approximately 80% of the variance of all PANDs consisted of five PCs (Fig. 2c) and suggested that NP has five major types of distribution around *cis*-regulatory elements in the genome. Therefore, we attempted to use these five PCs as five NP patterns to characterize nucleosome distribution around a TF as follows: w_1_ PC1 + w_2_ PC2 + … + w_5_ PC5, where PCi is the pattern *i* and w_i_ is the weight of pattern *i*. For example, MYOD_Q6 = −0.093 PC1 − 0.949 PC2 + 0.088 PC3 - 0.093 PC4 + 0.010 PC5. This demonstrated that the five NP patterns alone could reconstruct 92.5% of the original PAND shape of MYOD_Q6. We further determined the composition of all PANDs (Supplementary Table S1a). We fixed the sign (+/−) of PCs to reflect nucleosome density; a positive direction indicates a high frequency of nucleosome positioning while a minus direction indicates a low frequency of nucleosome positioning. When a PC score was negative, the vertically flipped shape of the PC, as shown in Fig. 2c, was observed in PANDs. We further evaluated if PCs are dependent on a specific data set by extracting PCs from MNase-treated data of C2C12 cells, generated by Asp *et al.* (Fig. 2d)^22^. As a result, we obtained five similar PCs and a sufficiently cumulative contribution ratio of the top five PCs (82.4%) (Supplementary Fig. S3a). The similarity of the five PCs between our data sets and the data sets of Asp *et al.* were assessed by calculating the degree with which the PCs of our data contained PCs from the data of Asp *et al.* (Supplementary Fig. S3b). Most PC1 and PC2 constituted a combination of PC1’ and PC2’ (where ’ indicates data from Asp *et al.*), PC3 contained >80% of PC4’, PC4 contained >90% PC3’ (PC3 and 4 were switched in the two studies) and approximately 80% of PC5 remained. Furthermore, we confirmed reproducibility within eight replicates (for C2C12 cells under growth [four replicates] and differentiation [four replicates] conditions). The five NP patterns were consistently captured (70–80%) in all replicates but the fifth PC was relatively less reproduced (~40%) (Supplementary Fig. S4). These results demonstrated the five PCs constitute the general NP pattern formed in mouse C2C12 cells.

PANDs from our data (Supplementary Fig. S2) have a spike at ±100 bp, and a similar shape was seen for the data from Asp *et al.*, although specific PCs were not extracted to explain this shape, either in our data or from the data of Asp *et al.* To address the cause of this sharp PAND shape, we assessed the sequence specific bias of MNase in PANDs because MNase has been shown to have A/T sequence digestion preference^25^,^26^. The proportion of nucleotides around the PPARA motif (PPAR response element) in myoblasts is plotted in Supplementary Figure S5a because the PPARA motif has a biased A/T sequence of 5′-GGNC**AAA**GC-3′ (Supplementary Fig. S5a). The A/T digestion preference was detected as the highest MNase signal spike at exactly 82 bp from the **AAA** position (Supplementary Fig. S5b; between the steep sided high G/C position). A similar spike was observed for the TATA motif (Supplementary Fig. S2). We therefore regarded the spike at ~100 bp as an artefact caused by sequence specific digestion that did not affect the extraction of the five NP patterns from MNase-Seq data.

### Shape characteristics of the five NP patterns

To understand each characteristic of the five NP patterns, we first determined the majority of NP in each NP pattern by extracting intensity (a) and position (b) of periodic signal having a certain frequency (c) by wavelet analysis. Each scalogram representation could be used for separating the major NP and for understanding the characteristics of a, b and c as follow.

#### Wide-trend NP (PC1)

**Figure 3:**
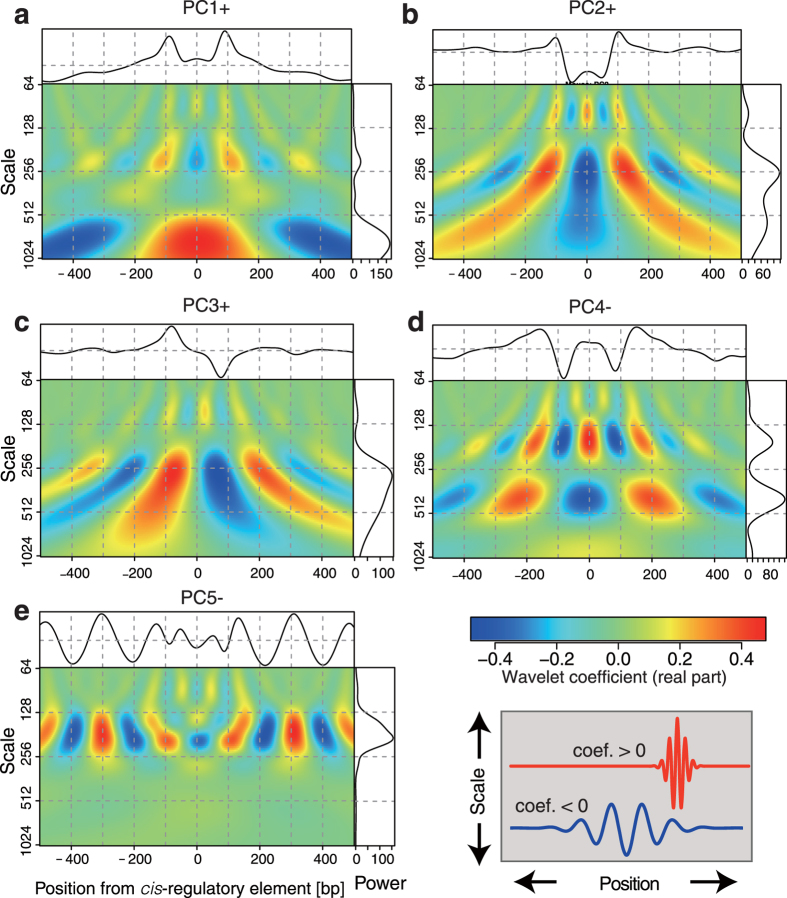
Five NP patterns characterized by frequency domain. Shape characteristics of five NP patterns were visualized by scalogram representation of wavelet coefficients. Each scalogram was labelled by PC name and the sign (vertical orientation) is shown in the wave shapes at the top of each box. The x-axis indicates the relative position ranges from −500 to +500 bp from a *cis*-regulatory element, and y-axis is the scale of a pseudo frequency of Morlet wavelet, ranging from 64-1024 Hz. The intensity of the real part of the wavelet coefficient is coloured from blue (negative) to red (positive), as shown in the colour-bar in bottom-right. The right box indicates the total power (x-axis) spectrum at each frequency (y-axis) of each PC in the window. The peak in the spectrum represents the major periodic component.

PC1 was mostly characterized by its ascending (PC1 score >0) or descending (<0) slope toward the centre. We plotted the scalogram of PC1+ (Fig. 3a). The spectral power (right box) in Fig. 3a represents “nucleosome occupancy” in >500 bp regions in PC1+, i.e. positioning is fuzzily determined with respect to the position of the *cis*-regulatory element. PC1− should be the vertically flipped shape of PC1+. PC1− could represent reduced nucleosome occupancy in the ~500 bp region around the centre. This is therefore the pattern that suggests broad nucleosome occupancy around a *cis*-regulatory element.

#### On-motif single NP (PC2)

PC2+ is mostly characterized by the deep dip ~200 bp wide at the centre (Fig. 3b). The almost flat shape beyond the dip represents less contribution to construct the PANDs shape in PC2. Therefore, we concluded the shape of PC2 represents a one “nucleosome configuration” on a *cis*-regulatory element, which is proposed terminology by Kaplan *et al.*^23^ since a single nucleosome only (<200 bp) can fit inside the dip. PC2− could represent the accumulation of a single nucleosome position on the *cis*-regulatory element.

#### Asymmetric NP (PC3)

PC3+ exhibited a point symmetric (line-asymmetric) shape as seen in the scalogram representation (Fig. 3c). PC3 can be explained by nucleosome positioning with a relatively long interval (>200 bp) and a unique nucleosome configuration with respect to a *cis*-regulatory element position. High PC3 scores were frequently found in the *cis-*regulatory elements of nuclear receptors (Supplementary Fig. S2) and could be explained by its unique positioning trend of the *cis*-regulatory element at the entry/exit site of nucleosomal DNA^27^. PC3− is the mirror-image positioning to PC3+ (vertically flipped shape). It should be noted that the asymmetry of PC3 followed the sequence orientation of *cis*-regulatory elements.

#### Protein-holding NP (PC4)

PC4− is associated with a shape in which nucleosomes are stably fixed at positions greater than the ordinary nucleosome repeat length (186 bp) from the binding site (Fig. 3d). The scalogram representation of PC4− shows that the NP has two mixed distributions (Fig. 3d). One shows a relatively short nucleosome repeat length (NRL) (frequency peaks at ~160 bp) around the centre of the 500 bp region. The other shows cyclic and wide-ranging occupancy (freq. at ~400 bp) around the centre. This mixed distribution in one PC can be understood if PC4− includes a factor-holding state between nucleosomes. This is supported by TF profiles showing that PC4− makes a higher contribution to the PANDs of ChIP-Seq data than to the PANDs of the 258 *cis-*regulatory elements (Supplementary Table S1a and S1b), suggesting that ChIP-Seq reports the actual binding site, not the *cis*-regulatory element, which contains information on both the TF-bound and unbound state. These results further suggest that PC4+ was not considered in the PANDs of both *cis-*regulatory elements and ChIP-Seq data because most PC4 scores were negative.

#### Regularly spaced NP (PC5)

The shape of PC5− exhibited a clear sinusoidal pattern with an <200 bp pitch (Fig. 3e). A large PC5− weight was seen at the CTCF binding site (Supplementary Table S1b), suggesting this pattern indicates a relationship with boundary formation on the genome or tightly packed nucleosome structure accompanying the NRL of <200 bp.

The shape characterizations of five patterns is consistent with the previous model of nucleosome positioning^28^. The NRL has been shown to be a periodic signal in MNase-Seq data, thus the periodic signal of 147–300 bp (>1 and <2 nucleosomal DNA size) is considered as nucleosome configuration in PC2, PC3 and PC5, while the periodic signal of >300 bp is considered as the occupancy pattern of PC1 and PC4. In conclusion, the occupancy pattern indicates fuzzily determined NP relative to the position of *cis-*regulatory elements along DNA, while the configuration pattern indicates exact positioning at *cis-*regulatory elements.

### Co-positioning of nucleosomes described in the five NP patterns

**Figure 4:**
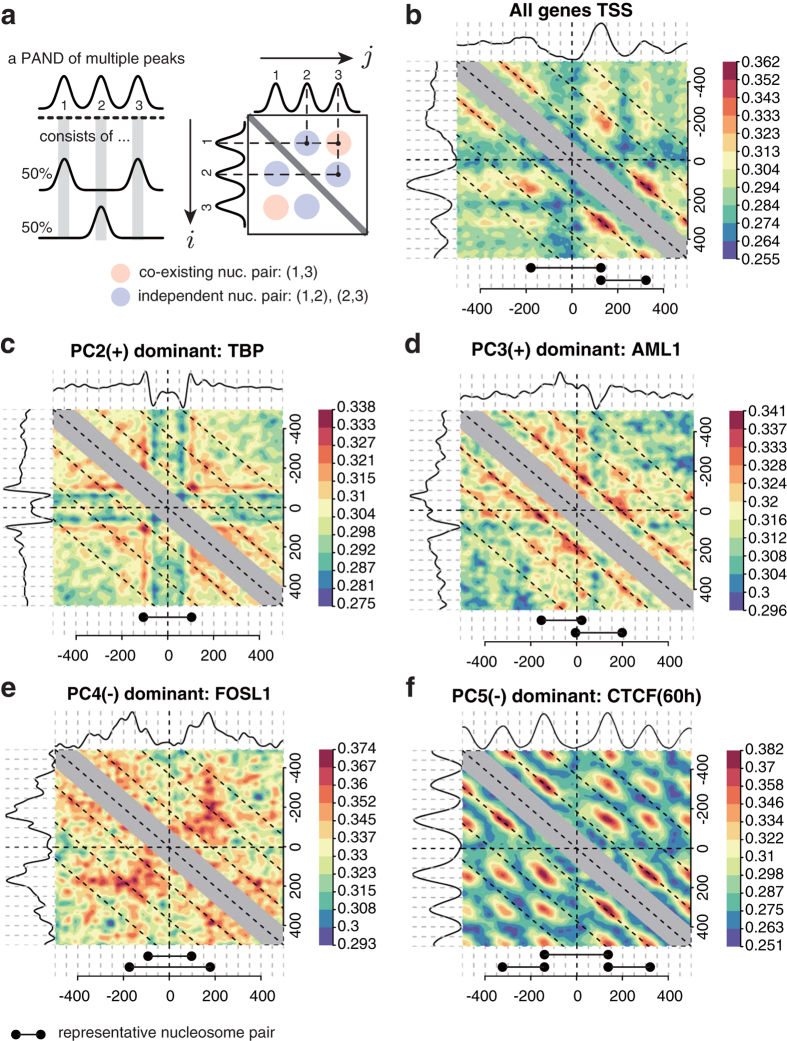
Frequently appearing nucleosome pairs. (**a**) Scheme of the nucleosome pair extraction method. A PAND with three peaks: 1, 2 and 3, which indicate three nucleosomes, is shown. However, the PAND is not sufficient for analyzing nucleosomes that appear at the same locus (**a**, left). Therefore, the degree of co-existence at two arbitrary points around an anchor-point was visualized (**a**, right). The pairs (1, 2) and (2, 3) indicate nucleosome pairs that appear independently, while the pair (1, 3) is a co-existing nucleosome pair at a single locus. (**b**–**f**) Examples of NPs based on all mouse genes’ TSSs and PC2-5, which show a representative *cis*-regulatory element or TF with high scores of each PC. The respective PAND shape is located at the top and left of each matrix. The colour-bars indicate the cosine similarity of a position pair in a matrix. Representative NPs are shown at the bottom of the matrices.

The shape characterization demonstrated that five NP patterns could describe NP on the genome. However, it was still unclear whether individual nucleosomes implied in the five NP patterns co-existed or were independent (Fig. 4a) because the shape of PANDs were averaged signals of NP on loci. Therefore, we attempted to confirm that a PAND having more than two peaks reflects a specific nucleosome configuration, as observed in PC2, PC4, and PC5, i.e. whether nucleosomes represented in the PAND’s peaks appear together at a single locus or not. The degree of co-existence was visualized by evaluating the cosine similarity of a nucleosome pair at all possible combinations of positions (Fig. 4b–f). We can visually inspect the peaks (spots) as co-positioned nucleosomes in a single locus. The data showed the co-existence (−1, +1) for a nucleosome pair and (+1, +2) nucleosome pairs at the TSSs of all mouse genes (Fig. 4b). The PC2 dominant NP pattern of the TBP *cis*-regulatory element (Fig. 4c) had less localization of the paired nucleosome at the centre. The PC3 dominant NP pattern showed the mirror image of co-existing nucleosome pairs (Fig. 4d). The PC4 dominant pattern (Fig. 4e) showed fuzzily determined positioning of pairs at approximately ±200 bp. The PC5− dominant pattern of the CTCF binding site in C2C12 cells (Fig. 4f) showed clear co-existing nucleosome pairs between all peaks. These results confirmed that PC1 reflected the frequency of independent single nucleosomes (nucleosome occupancy) while PC2, PC4 and PC5 reflected specific configurations of co-existing nucleosomes represented by multiple peaks in the PCs.

### Correlation between five NP patterns and gene expression

**Figure 5:**
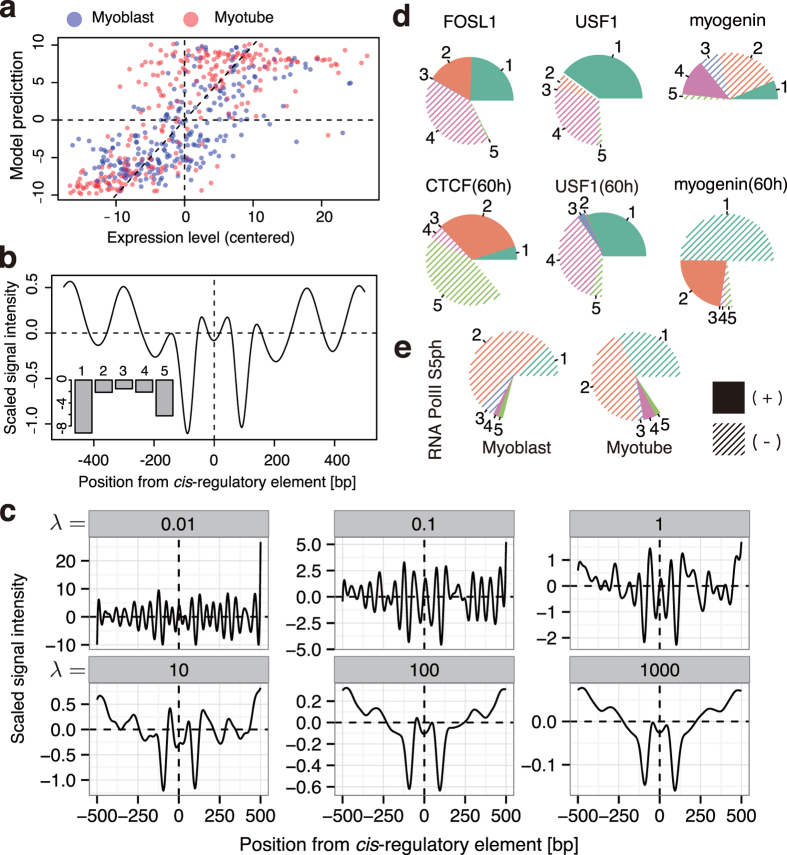
Functional profile of the five components. (**a**) PCR model-predicted gene expression levels. The x-axis indicates centred average gene expression level associated with each of the 258 *cis-*regulatory elements. The y-axis indicates gene expression level by PCR model. Blue and red points indicate myoblasts and myotubes, respectively (n = 512). (**b**) The wave shape and its PC weights of the best NP pattern for gene expression prediction by PCR are shown. The x-axis indicates relative position from the *cis*-regulatory element (the centre is at x = 0), the y-axis indicates scaled and centred MNase-Seq signal intensity. The left-bottom bars represent the weights of PC1-5 (labelled as 1–5) of the wave shape. (**c**) The best NP pattern for predicting gene expression derived by ridge regression analysis. The six boxes show the result of the best NP patterns with various bias parameters and *λ* values. The *λ* values are shown in the head of each box. The x and y-axes are the same as in (**b**). (**d**) Functional profile of ENCODE TFBS data in mouse C2C12 cells. The weight of each component is presented as a pie chart where the composition ratios (%) of the five components are indicated. Numbers correspond to PC1-5. Composition ratios from components other than PC1–5 are shown as blank regions. (**e**) Functional profiling of RNAP2-Ser5ph in myoblasts/myotubes shown in (**d**).

To address how the five NP patterns correlate with gene expression, we attempted to determine ideal NP patterns, composed of the five NP patterns, around expressed genes. We then predicted gene expression by ideal NP consisting of the five NP patterns. We performed two independent regression analyses as follows. The first method simply determined the best ratio of the five NP patterns to predict gene expression. The task was formulated as principal component regression (PCR) analysis that was slightly modified for predicting gene expression using PC scores as follows:


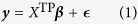


where ***y*** is a vector of the averaged neighbouring gene expression within 2 Kbp from each *cis*-regulatory element, *X* is a matrix of PANDs and *P* is a matrix of which columns consists of the five PC vectors, i.e. *X*^T^*P* becomes the PC score matrix. The least square (LS) estimator of *β* minimizes ||
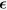
||^2^ was derived as follows:


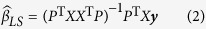


which led to the result shown in Fig. 5a, and the coefficient of determination *R*^2^ = 0.51, which indicates that >50% of the variance in the gene expression around the *cis-*regulatory elements were explained (Pearson’s correlation coefficient between the predicted expression level 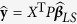 and ***y*** was 0.72, and Spearman correlation was 0.74; Fig. 5a). This ideal NP predicted by PCR was drawn by calculating 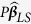 (Fig. 5b). The major component of the ideal NP showed highly weighted PC1 and limited weight of PC5 (Fig. 5b; bottom-left). These results suggest that gene expression was correlated with the NP pattern of descending nucleosome occupancy (PC1−) and regularly spaced nucleosomes (PC5−).

To clarify the contribution of each PC for predicting gene expression, we assessed the correlation between gene expression level and each PC score. PC1− correlated with expression levels while PC1+ negatively correlated with gene expression. In the case of PC5, only PC5+ negatively correlated with expression levels (Supplementary Fig. S6; only the 4^th^ quadrant of gene expression levels had a linear relationship to the PC5 score). Other PCs did not show substantial correlation. These results revealed that PC1+/− and PC5+ contribute to the gene expression predicted in Fig. 5a,b.

The second method we used calculated ideal NP for transcription directly, which is independent from the five NP patterns. To determine the most contributing NP to gene expression, ***y***, the best NP pattern, *β*, should meet the following relationship:


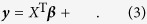


This system would be over-determined (#*cis-*regulatory elements < #columns of *X*^T^), therefore we used ridge regression by adding λ as a bias parameter to avoid the over-fitting problem. The ridge estimator of *β* was calculated as follows:


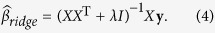


The ideal NP pattern was plotted as *β* in Fig. 5c using various *λ* (from 10^−2^ to 10^3^). The weights of the *β* were calculated by dot product against the five NP patterns in the settings of *λ* (Supplementary Fig. S7). The result demonstrated that ridge regression gave the ideal NP and that PC1 and PC5 were the critical components for expression level, which is consistent with the PCR analysis.

We conclude from these two independent approaches that the ideal NP pattern for gene expression had a large valley-like occupancy trend (PC1−) with regular spacing (PC5−) NP and gene expression could be predicted by the context of PC1 and PC5.

### Tracking chromatin state changes at TFBSs using the five NP patterns

Because gene expression could be predicted by the context of the five NP patterns, we further attempted to analyze TF function as an application of our five NP patterns. We first generated PANDs around various intact TFBSs obtained from ChIP-Seq data of different cell conditions deposited in ENCODE projects (Fig. 5d)^29^, and then decomposed the PANDs into the five NP patterns (Supplementary Table S1b). The profile of FOSL1 showed large PC1+ and PC4− in myoblasts (unlabelled; 0 h), and USF1 showed a similar and consistent profile in both myoblasts and myotubes (60 h). These results were consistent with constitutive binding of FOSL1 and USF1 and their transcriptional suppression in differentiation^30^. Additionally, FOSL1 is reported to have a bZIP motif that can activate transcription^30^; thus when coupled with the AP-1 family, PC2+ in FOSL1 indicates that FOSL1 could be involved in transcription activation. On the other hand, myogenin showed changes in its weights during differentiation (Fig. 5d). The PAND of myogenin at 0 h was not well characterized by the five PCs (i.e. half of the compositions were blank) because myogenin is not expressed in the myoblast, which reflects the fact that myogenin at 0 h did not contribute to transcription activation. However, PANDs had large composition rates for PC1− and PC2+ at 60 h, which suggests an active transcription state. These results are consistent with previous reports of TFs in myogenic differentiation^31^, and indicate that this approach can be useful for predicting TF function as well as gene expression level. We further attempted to validate the approach using in-house ChIP-Seq data. ChIP-Seq on the RNAP2 phosphorylated carboxyl-terminal domain (CTD) serine-5 (RNAP2-S5ph) was performed using MNase digestion following fixation. After identifying RNAP2-S5ph loading sites on the genome, PANDs around RNAP2-S5ph were profiled by the composition of the five PCs. The shapes of the PANDs for the myoblast and myotube were mostly due to PC1 and PC2, with PC1−weights being much higher in the myotube than in the myoblast stage (Fig. 5e). Because our RNAP2-S5ph antibody recognizes both the poised state (S5ph) and the active transcribing state (S5ph + S2ph)^32^,^33^, the increase in PC1− suggests an increase in transcriptional activation around the RNAP2-S5ph binding sites, which is consistent with a previous finding that showed increased transcription of activated genes during skeletal muscle differentiation^34^. Therefore, these data suggest that the decomposition of a PAND to the five NP patterns could also be used to follow changes in TF function.

## Discussion

Here we identified five major NP patterns on the mouse genome that were correlated to gene expression or specific types of TF binding sites. These results suggest that NP patterns act as functional units on chromatin and describe the transcriptional state of the TFs. Similarly, chemical modifications of histones or RNAP2 have been reported as histone or CTD codes that can describe the transcription regulation state^35^,^36^,^37^. Further functional elucidation of these codes and their combination could lead to complete descriptions of chromatin structure.

Our method assumes that most nucleosome-wrapping DNAs produce mono-nucleosome sized DNA fragments. However, some fragments, such as small histone alternative proteins, non-nucleosomal proteins or poly-nucleosomes may produce variable sized fragments^19^. Furthermore, MNase-Seq data include various types of nucleosomes with non-canonical histone variants, such as H2A.Z and H3.3. Further understanding of how complex NP conformations contribute to transcription regulation requires more structural characterization of NP, such as analysis of the histone variants associated with the nucleosome, as this would provide more information on the flexibility of the DNA-nucleosome interaction, the co-localizations of the histone variants on the genome and the contribution of histone modifications to gene expression.

Another limitation of our method is that the evaluation depends on averaging, and NP evaluation did not focus on individual NP at single loci in single cells. Recently, a computational reconstruction method of positioning of a single nucleosome from observed MNase-Seq signals at a single gene locus was proposed^38^. Although this method could estimate NP at each single site on the genome, a large number of reads was required to recapitulate the single NP level. In our case, to confirm NP formed by poly-nucleosomes at single loci, we evaluated the co-existence of nucleosomes statistically (Fig. 5). Our finding could be confirmed by further approaches that examine the spatial arrangements of poly-nucleosomes at the single cell level by determining each nucleosome position at a single locus.

Technically, previous MNase-Seq analysis has used native chromatin with various MNase digestion conditions. Here we used a chromatin fraction that was fixed and then digested with MNase, which resulted in pure mononucleosome-sized DNA fragments. The fragment size might be considered to be over-digested^39^. However, even if chromatin was over-digested in generating the MNase-Seq data, any effect is likely to be minimal because Rando *et al.* showed this produced a minor effect in drawing PANDs.

Finally, we demonstrated that our approach can be applied to the ENCODE data of C2C12 cells, which suggests its applicability to other ChIP-Seq data for the prediction of TF function in transcription regulation. Although this study focused on TFs, the same method could be applied to specific histone modifications or other epigenetic markers on the genome. However, to better understand the function of transcription-independent NP patterns, such as narrow-ranged, asymmetric and protein-holding patterns, other functional predictions and understanding of other NP patterns are needed to study higher-order chromatin structures.

## Materials and Methods

### Data access

Fixed MNase-Seq data and ChIP-Seq data of RNAP2-S5ph in myoblast/myotube cells were submitted to the DDBJ Sequence Read Archive with the accession number, DRA001262.

### Cells

C2C12 cells were cultured in Dulbecco’s modified Eagle’s medium (DMEM) supplemented with 20% foetal bovine serum. Cells examined under growth conditions (myoblasts) were harvested at 60–70% confluency. Differentiated samples (myotubes) were transferred to DMEM containing 2% horse serum upon reaching confluence and harvested 48 h later.

### MNase digestion, chromatin immunoprecipitation and deep sequencing

Cells were fixed with 0.5% formaldehyde for 5 min at room temperature, and then blocked with 150 mM glycine, pH 7.0. After sonication (three 5 sec pulses), the cells were digested with MNase for 40 min at 37 °C. DNA fragments of approximately 200 bp were selectively purified following 1% TAE agarose gel electrophoresis. Library preparation was performed as described in Odawara *et al.*^32^. ChIPed DNA was sequenced on an Illumina HiSeq 1500 platform (Illumina, San Diego, CA, USA), and the obtained reads were mapped to the mouse genome (mm9) using Bowtie2 software (version 2.1.0) with default parameter settings. Multi-hit reads were discarded. The MNase-seq signal averaging (aggregation) was calculated using our agplus^40^ software. The binding sites of RNAP2-S5ph were obtained with MACS (version 2.0.10.20130528) using the options “–*gsize mm* –*nomodel* –*extsize (average ChIPed DNA fragment size, 170 for myoblast and 159 for myotube)* –*broad* –*to-large* –*pvalue 1e-3*”.

### Density estimation of the nucleosomes

PANDs were created by linear convolution of nucleosome mid-point counts as follows:


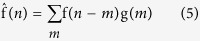


where f(*x*) is the RPM (read per million) normalized count of the nucleosome mid-point from the relative distance *x* (in bp) using the reads per million normalization^41^. We used [−500,500] as the range of *x* from a *cis*-regulatory element centre. g(*x*) is a Gaussian kernel,


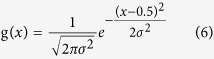


where σ is the bandwidth parameter. We calculated the convolution over the range *m* ∈ [*−4σ, 4σ*], because g(*x*) becomes sufficiently small when | *x* | >4*σ*. The term *x – 0.5* is a continuity correction.

### Genomic region definitions of *cis-*regulatory elements

258 *cis*-regulatory element-containing region definitions were acquired from the University of California Santa Cruz (UCSC) Transcription Factor Binding Site (TFBS) Conserved Track (*tfbsConsSites*). The *tfbsConsSites* track defines *cis*-regulatory element-containing regions from the Transfac Matrix Database (v7.0) of the human genome (hg19). The tfbsConsSites for the mouse genome were obtained by comparing conservation between human and mouse genomes (mm9) using UCSC’s *liftOver* tool. We used the transRegCode database^42^ for the definition of *cis-*regulatory elements in the yeast (*S. cerevisiae*) genome.

### ENCODE ChIP-Seq TFBS data

TFBSs were acquired by ChIP-Seq from mouse C2C12 cells using ENCODE/Caltech (GSE36024) and used to profile TF function with PANDs. TFBSs calculated from the data were obtained using *narrowPeak* from UCSC:

http://hgdownload.cse.ucsc.edu/goldenpath/mm9/encodeDCC/wgEncodeCaltechTfbs.

### Principal component analysis (PCA) of average density profiles

A matrix, *X*, of 1001 × 516 elements was generated. Columns contain PANDs for 258 *cis-*regulatory elements ±500 bp at a 1 bp resolution (516 vectors of length 1001). Both myoblast and myotube conditions were contained (258 + 258 = 516). The matrix constructed from the data of Asp *et al.*^22^ also contained both myoblast and myotube states and their replicates. Each column of *X* was normalized to have an average = 0 and Euclidean norm = 1. This normalization was used to ensure the sum of the squared score of all principal components (PCs) was 1 (all score vectors on the surface of a unit hypersphere). The eigenvectors of the covariance matrix, *1/(516-1) XX*^*T*^, were used as the PCs, and the cumulative contribution ratio was computed from the cumulative sum of eigenvalues.

### Wavelet analysis

Wavelet analysis was performed using the Morlet wavelet defined as:


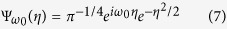


where we used the parameter 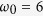. We calculated inner-product between a NP pattern p(*x*) and 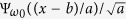 at scale *a* ∈ [*64,1024*] and localized position *b* ∈ [*−500, 500*]. To capture both the frequency and the peak localization of the wave shape, we showed only the real part of the wavelet coefficients in scalogram representation. The total power spectrum density in a window was calculated using the real and imaginary part. We made our wavelet analysis demo using R in Supplementary File 1.

### Gene expression analysis

Gene expression data of C2C12 cells were obtained from DRA000457 in the DDBJ Sequence read archive. Genes were associated with a *cis*-regulatory element when the *cis*-regulatory element existed within 2 kbp of a gene. The number of associated genes was 19,703 out of all refFlat mm9 genes. Approximately 6,000 genes (min: 883, max: 10,379, mean: 6,271, s.d.: 1,578) were associated to each *cis*-regulatory element. To assign gene expression levels to each *cis*-regulatory element, the average expression levels were calculated for each associated gene set.

### Extraction of frequently appearing nucleosome pairs

The co-existence of nucleosomes detected simultaneously at two arbitrary points around an anchor-point was computed using smoothed density profiles at individual sites. We defined *A* as a matrix that holds all combinations of two arbitrary points within ±500 bp of the anchor-point [e.g. transcription start site (TSS) or centre of the *cis*-regulatory element]. Its elements are cosine similarities for all combinations of *i, j* = *−500, −499, …, 499, 500*, where the cosine similarity is defined as


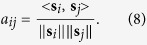


The vectors ***s***_*i*_ and ***s***_*j*_ are the *i*-th and *j*-th columns of PANDs matrix *S*, an *M* × *N* matrix, where *M* is the total number of individual sites and *N* is the range (in bp) of the nucleosome density. We set *N* = *1001*, i.e. ±500 bp from the anchor-point. The notation 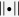 is a Euclidean norm (root sum squared), and the function <, > is the dot product of vectors. Thus *a*_*ij*_ measures the angle between ***s***_*i*_ and ***s***_*j*_. We made the co-positioning nucleosome analysis demo using R in Supplementary File 1.

## Additional Information

**How to cite this article**: Maehara, K. and Ohkawa, Y. Exploration of nucleosome positioning patterns in transcription factor function. *Sci. Rep.* **6**, 19620; doi: 10.1038/srep19620 (2016).

## Supplementary Material

Supplementary Figures

Supplementary Tables
