# Supplementary material for: Exploration of nucleosome positioning patterns in transcription factor function
Source: Sci Rep. 2016 Jan 21;6:19620. doi: 10.1038/srep19620 (PMC4726364; doi:10.1038/srep19620)
Supplement: Supplementary Figures [file srep19620-s1.pdf]

SUPPLEMENTARY FIGURES

Exploration of nucleosome positioning patterns in transcription factor function

Kazumitsu Maehara[1] and Yasuyuki Ohkawa[1]*

[1]Department of Advanced Medical Initiatives, JST-CREST, Faculty of Medical Sciences, Kyushu University, 3-1-1 Maidashi, Higashi-ku, Fukuoka, Fukuoka 812-8582, Japan.

*To whom correspondence should be addressed. Tel: +81-92-642-6216; Fax: +81-92-642-6099; Email: yohkawa@epigenetics.med.kyushu-u.ac.jp

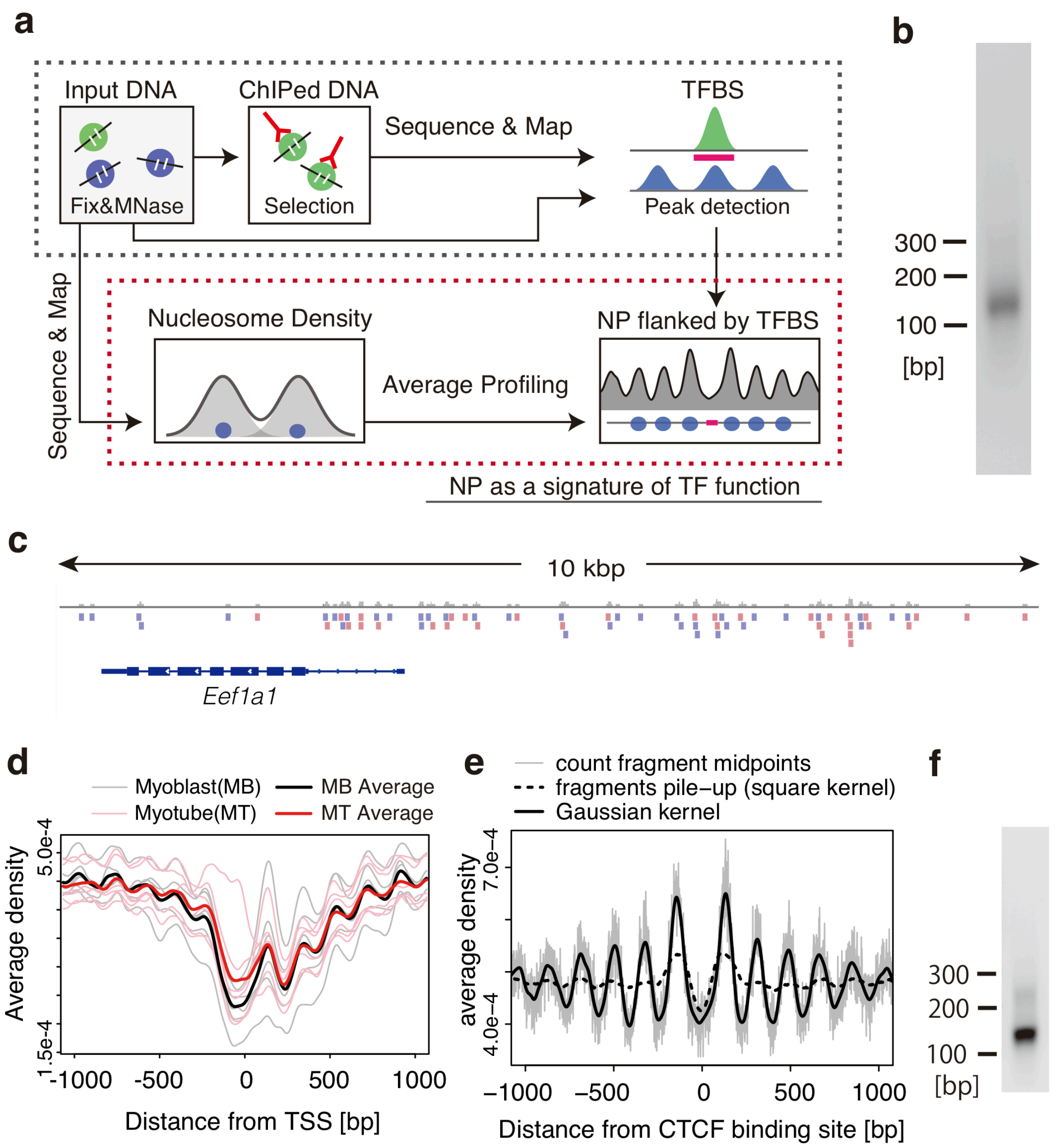

Figure S1 | Nucleosome density estimates.

(a) Schematic depiction of the work flow for average nucleosome density estimation. First, chromatin is fixed and digested by MNase to obtain mono-nucleosome sized DNA fragments. The samples are deproteinized, deep sequenced and the DNA mapped onto the reference genome. Next, the nucleosome detection frequency is averaged at all defined anchor points (e.g. TSSs, TFBSs). Finally, NP is predicted by the smoothed density profile of the nucleosomes.

(b) DNA fragment size of the ChIP-Seq (RNAP2-S5ph) samples. DNA fragments of mono-nucleosome size (164 ± 12 bp; mean ± standard deviation) were obtained.

(c) Mapped sequence reads around the *Eef1a1* gene locus. An example of mapped deep sequence reads of fixed MNase-Seq at the *Eef1a1* locus is shown. The blue/red boxes indicate the forward/reverse stranded mapped reads, respectively, on the mouse genome. **(d)** Nucleosome densities around TSSs determined by Asp et al. using Mnase-digested input control DNA from ChIP-Seq analysis of C2C12 cells. Myotube and myoblast replicates (thin pink/grey lines) are shown together. Each average density is shown as bold red/black lines. **(e)** Nucleosome densities around the CTCF binding site. Comparisons of NP estimates using a simple count of the fragment mid-point (grey), fragments pile-up (dotted), or smoothed fragment mid-point by a Gaussian kernel (solid). TFBS data were obtained from the ENCODE project. **(f)** DNA fragments of non-fix samples were of mono-nucleosome size (154 ± 11 bp).

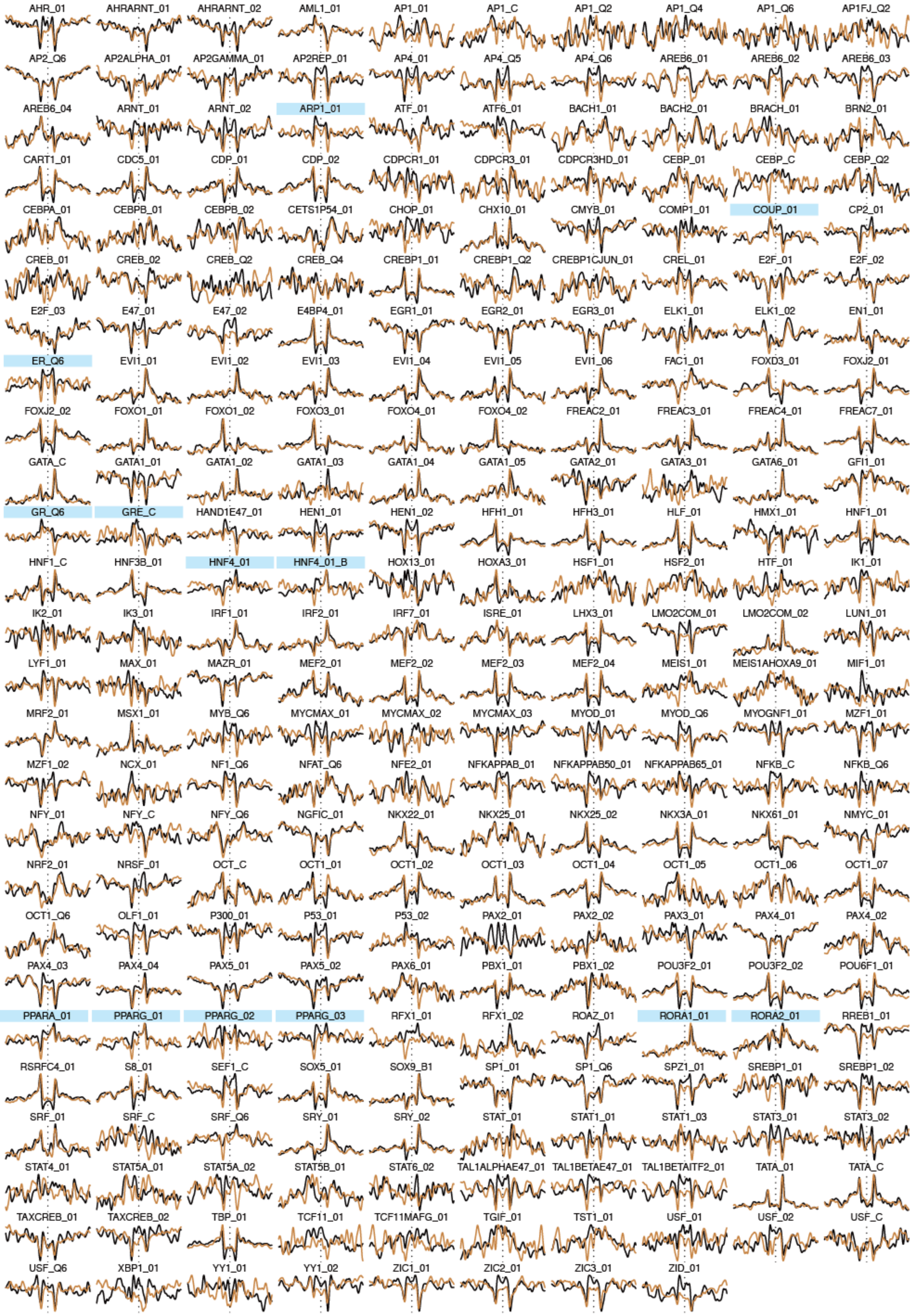
AHR_01
AHRARNT_01
AHRARNT_02
AML1_01
AP1_01
AP1_C
AP1_Q2
AP1_Q4
AP1_Q6
AP1FJ_Q2
AP2_Q6
AP2ALPHA_01
AP2GAMMA_01
AP2REP_01
AP4_01
AP4_Q5
AP4_Q6
AREB6_01
AREB6_02
AREB6_03
AREB6_04
ARNT_01
ARNT_02
ARP1_01
ATF_01
ATF6_01
BACH1_01
BACH2_01
BRACH_01
BRN2_01
CART1_01
CDC5_01
CDP_01
CDP_02
CDPCR1_01
CDPCR3_01
CDPCR3HD_01
CEBP_01
CEBP_C
CEBP_Q2
CEBPA_01
CEBPB_01
CEBPB_02
CETS1P54_01
CHOP_01
CHX10_01
CMYB_01
COMP1_01
COUP_01
CP2_01
CREB_01
CREB_02
CREB_Q2
CREB_Q4
CREBP1_01
CREBP1_Q2
CREBP1CJUN_01
CREL_01
E2F_01
E2F_02
E2F_03
E47_01
E47_02
E4BP4_01
EGR1_01
EGR2_01
EGR3_01
ELK1_01
ELK1_02
EN1_01
ER_Q6
EVI1_01
EVI1_02
EVI1_03
EVI1_04
EVI1_05
EVI1_06
FAC1_01
FOXD3_01
FOXJ2_01
FOXJ2_02
FOXO1_01
FOXO1_02
FOXO3_01
FOXO4_01
FOXO4_02
FREAC2_01
FREAC3_01
FREAC4_01
FREAC7_01
GATA_C
GATA1_01
GATA1_02
GATA1_03
GATA1_04
GATA1_05
GATA2_01
GATA3_01
GATA6_01
GFI1_01
GR_Q6
GRE_C
HAND1E47_01
HEN1_01
HEN1_02
HFH1_01
HFH3_01
HLF_01
HMX1_01
HNF1_01
HNF1_C
HNF3B_01
HNF4_01
HNF4_01_B
HOX13_01
HOXA3_01
HSF1_01
HSF2_01
HTF_01
IK1_01
IK2_01
IK3_01
IRF1_01
IRF2_01
IRF7_01
ISRE_01
LHX3_01
LMO2COM_01
LMO2COM_02
LUN1_01
LYF1_01
MAX_01
MAZR_01
MEF2_01
MEF2_02
MEF2_03
MEF2_04
MEIS1_01
MEIS1AHOXA9_01
MIF1_01
MRF2_01
MSX1_01
MYB_Q6
MYCMAX_01
MYCMAX_02
MYCMAX_03
MYOD_01
MYOD_Q6
MYOGNF1_01
MZF1_01
MZF1_02
NCX_01
NF1_Q6
NFAT_Q6
NFE2_01
NFKAPPAB_01
NFKAPPAB50_01
NFKAPPAB65_01
NFKB_C
NFKB_Q6
NFY_01
NFY_C
NFY_Q6
NGFIC_01
NKX22_01
NKX25_01
NKX25_02
NKX3A_01
NKX61_01
NMYC_01
NRF2_01
NRSF_01
OCT_C
OCT1_01
OCT1_02
OCT1_03
OCT1_04
OCT1_05
OCT1_06
OCT1_07
OCT1_Q6
OLF1_01
P300_01
P53_01
P53_02
PAX2_01
PAX2_02
PAX3_01
PAX4_01
PAX4_02
PAX4_03
PAX4_04
PAX5_01
PAX5_02
PAX6_01
PBX1_01
PBX1_02
POU3F2_01
POU3F2_02
POU6F1_01
PPARA_01
PPARG_01
PPARG_02
PPARG_03
RFX1_01
RFX1_02
ROAZ_01
RORA1_01
RORA2_01
RREB1_01
RSRFC4_01
S8_01
SEF1_C
SOX5_01
SOX9_B1
SP1_01
SP1_Q6
SPZ1_01
SREBP1_01
SREBP1_02
SRF_01
SRF_C
SRF_Q6
SRY_01
SRY_02
STAT_01
STAT1_01
STAT1_03
STAT3_01
STAT3_02
STAT4_01
STAT5A_01
STAT5A_02
STAT5B_01
STAT6_02
TAL1ALPHAE47_01
TAL1BETAE47_01
TAL1BETAITF2_01
TATA_01
TATA_C
TAXCREB_01
TAXCREB_02
TBP_01
TCF11_01
TCF11MAFG_01
TGIF_01
TST1_01
USF_01
USF_02
USF_C
USF_Q6
XBP1_01
YY1_01
YY1_02
ZIC1_01
ZIC2_01
ZIC3_01
ZID_01

Figure S2 | PANDs of 258 TRANSFAC *cis*-regulatory elements.

PANDs of +/-500 bp from the *cis*-regulatory element center of 258 types of TFBS. Coordinate axes are omitted to show only the shapes of the PANDs. Black and brown lines indicate myoblast and myotube PANDs, respectively. The labels highlighted in blue indicate nuclear receptors.

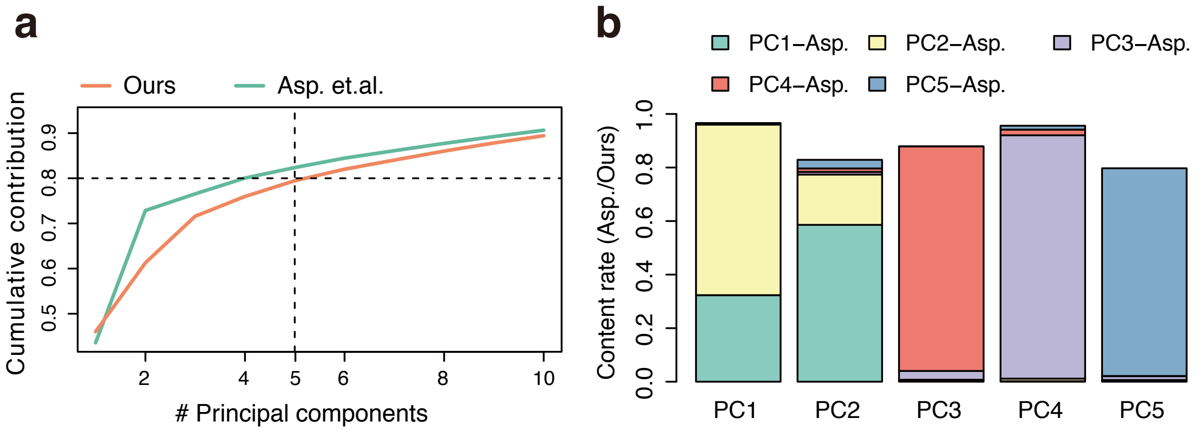

Figure S3 | Similar NP patterns were extracted from different data set of mouse C2C12 cells

(a) The top five PCs reached a ~80% cumulative contribution rate. The x-axis is the number of PCs used and the y-axis is the cumulative contribution rate. The orange line is our MNase-Seq data and the green line is from the data of Asp et al. The dotted lines indicate five PCs (vertical) and the contribution rate of 0.8 (horizontal).

(b) The similarity between PCs was measured by dot product between PC vectors from our data (PC1-5) and the data of Asp et al. (PC1'-5'). Each height of a vertical bar labelled PC1-5 indicates the sum of squared values of dot product (max: 1.0) between PC1 vs. PC1'-5', PC2 vs. PC1'-5' and so on.

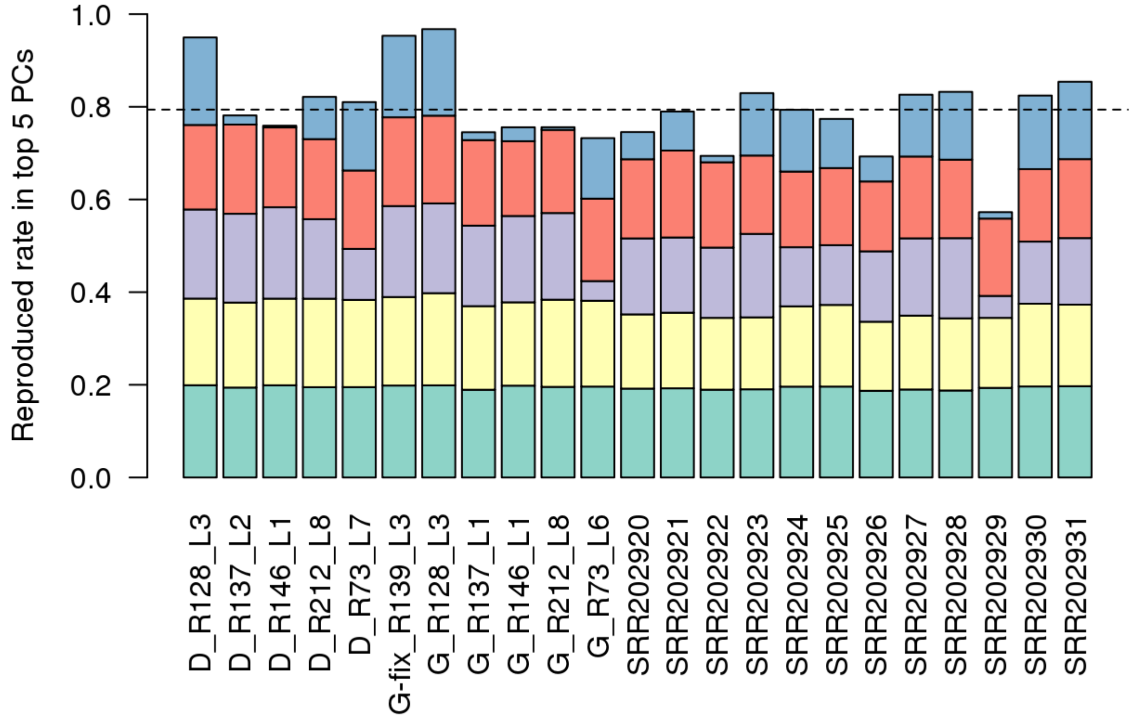

Figure S4 | Reproducibility of five PCs

The reproducibility of the five NP patterns using replicates of MNase-seq data is shown as series of stacked bar plots. The different colours of the stacked bars represent the reconstruction ratio (max=0.2) of PC1-5 from bottom to top. The reconstruction ratio was calculated by applying the inter-product of the five PCs and each reproduced PC1-5. The replicates were for C2C12 cells under growth (four replicates) (prefix “G”; middle part of the bars) and differentiation (four replicates) conditions (prefix “D”; left part) and Asp. data (with “SRR” prefix). G_R128, D_128 were used in main results and G-fix was also used in Figure 1b.

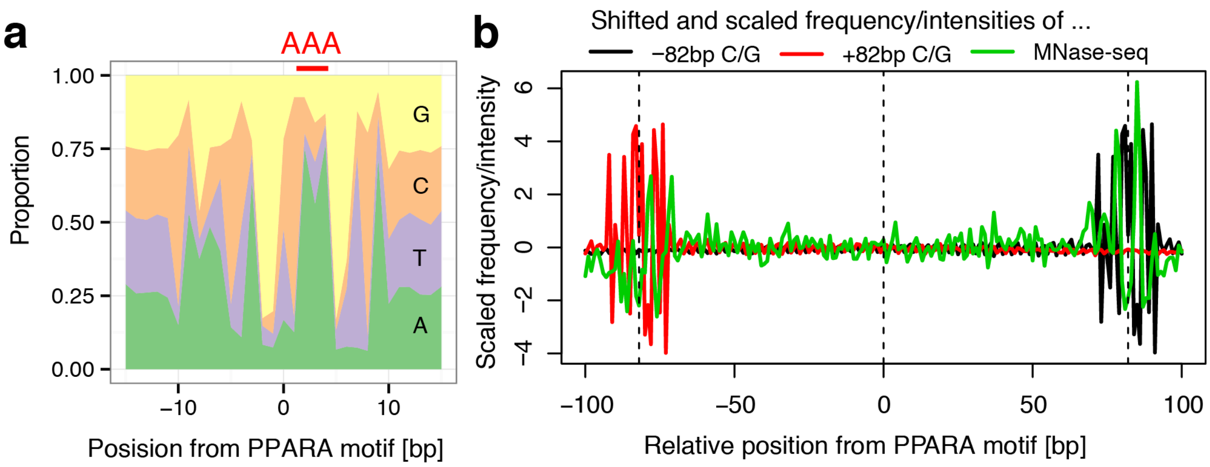

Figure S5 | DNA sequence bias makes pseudo signal peak in MNase-Seq data

(a) The observed ratio of each nucleotide around the PPARA motif in the mouse genome. The x-axis indicates relative position from the PPARA motif center, ranging from -15 to +15 bp. The y-axis indicates proportion of observed nucleotides. The colour of the area indicates each nucleotide (G: yellow, C: orange, T: purple, A: green). The position of the highly biased AAA sequence that has high MNase digestion preference is marked at the top in red.

(b) The A/T digestion bias produces the spiky artifact seen in the MNase-Seq data. The MNase–Seq data of the C2C12 myoblast state was used for this example. The x-axis indicates relative position from the PPARA motif. To compare signals that have different scales, the y-axis is shown as centerd and scaled (mean=0, s.d.=1) signal intensities/frequency of the nucleotide frequency of C/G. The black and red lines are the +82 or -82 bp-shifted C/G sequence frequency. The shift was the requirement for estimating nucleosome center. The highest MNase-Seq signal spikes appeared just between the shifted high C/G biased point (AAA).

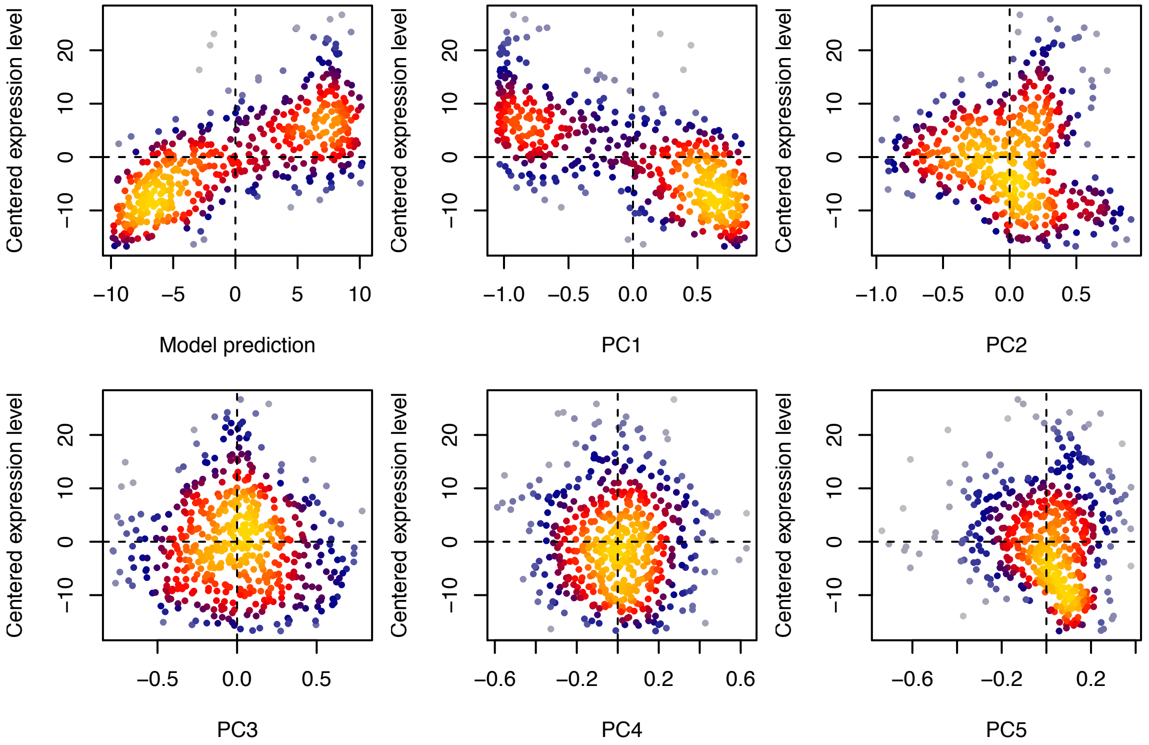

Figure S6 | PC1 is a critical component to predict gene expression

The scatterplots of PC scores and the scaled and centerd average gene expression values are shown. The x-axis of first box (top-left) indicates predicted expression level by PCR, and the others indicate PC scores of each PC. The y-axis is the scaled and centerd gene expression level. The density of the points is represented by colours, from grey (lowest), through blue, red, orange to yellow (highest).

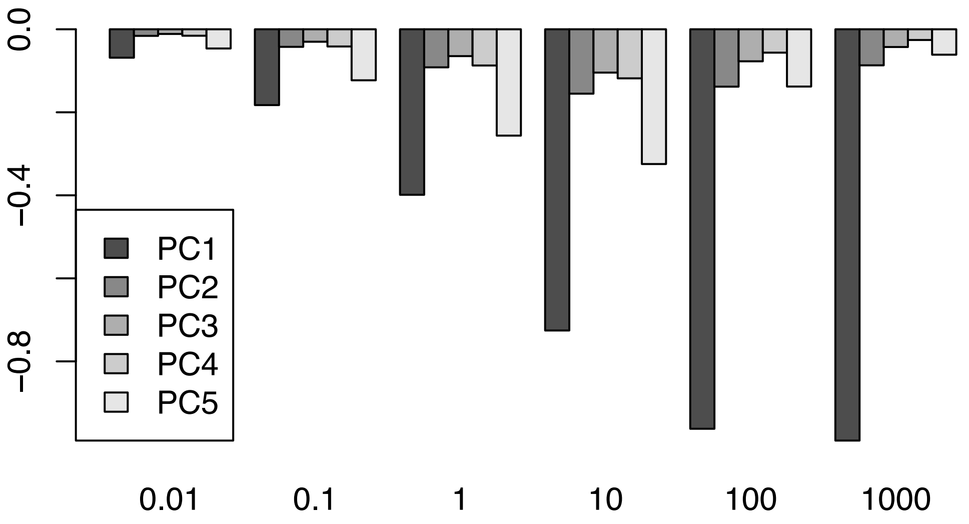

Figure S7 | Ideal NP pattern for gene expression consists of PC1 and PC5

The weights of five the PCs contained in the ideal NP pattern for gene expression derived by ridge regression analysis are shown. The height of bars (y-axis) indicates the dot product calculated between five PCs and each ideal NP pattern of λ. The bottom labels indicate the λ values used for each ridge regression model. The bars in each λ are in the order PC1-5 (black-grey).
